# Scientometrics and meta-research in medical research: approaches required to ensure scientific rigor in an era of massive low-quality research

**DOI:** 10.1590/1806-9282.20241612

**Published:** 2025-06-06

**Authors:** Ivan David Lozada-Martinez, David Hernandez-Paez, Yesenia Esther Jiménez Zárate, Patricia Delgado

**Affiliations:** 1 Universidad de la Costa, Department of Health Sciences, Biomedical Scientometrics and Evidence-Based Research Unit – Barranquilla, Colombia.; 2 Centro de Meta-Investigación y Cienciometría en Ciencias Biomédicas – Barranquilla, Colombia.; 3 Universidad de Cartagena, Grupo Prometheus y Biomedicina Aplicada a las Ciencias Clinicas, School of Medicine – Cartagena, Colombia.; 4 Universidad de la Costa – Barranquilla, Colombia.; 5 Universidad Nacional Autónoma de Nicaragua – Managua, Nicaragua.

The post-pandemic era caused by coronavirus disease-2019 (COVID-19) has made a previously reported trend more visible over the last two decades: the "avalanche" of biomedical publications. One of the greatest paradoxes in contemporary science is the increasing availability of scientific evidence, coupled with a decrease in its quality. This has been a topic of broad international discussion, involving ethical, methodological, social, economic, and political arguments. In recent years, there has been a significant increase in paid journals, which has raised concerns about the quality of research being published, particularly in the context of government-funded projects. These journals often prioritize revenue generation over scientific rigor, creating a platform for studies with questionable methodologies to gain visibility. The pressure on researchers to publish frequently, driven by academic and institutional evaluation systems, has further exacerbated this issue. Also, the rise of predatory journals represents a critical challenge in maintaining the integrity of biomedical literature. These journals operate outside conventional editorial frameworks, often bypassing the peer-review process, which is a cornerstone of scientific credibility. This phenomenon has been fueled by the proliferation of open-access publishing models, which, while democratizing access to scientific findings, have also created opportunities for exploitative practices.

**Figure 1:**
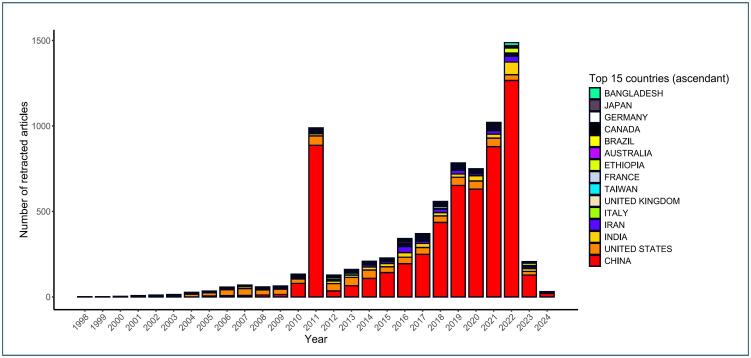
Top 15 countries with the highest frequency of retractions in the field of medical and health sciences over time.

**Figure 2:**
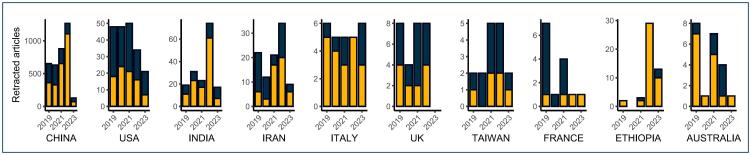
Retractions according to funding status in the top 10 countries with the highest frequency of retractions (yellow: no funding vs. black: funded).

These discussions have converged on a common conclusion: the threat to scientific rigor and the impact of using erroneous or fraudulent data in the worst scenario, real-life contexts. An illustrative example is the significant number of retractions and complaints about scientific misconduct by both journals and researchers, particularly notable in the field of medical research (Figure 1). Based on a brief bibliometric analysis using the Scopus database and the specific search strategy "DOCTYPE (tb) AND [LIMIT-TO (SRCTYPE, "j") OR LIMIT-TO (SRCTYPE, "p")] AND [LIMIT-TO (SUBJAREA, "MEDI") OR LIMIT-TO (SUBJAREA, "BIOC") OR LIMIT-TO (SUBJAREA, "IMMU") OR LIMIT-TO (SUBJAREA, "HEAL") OR LIMIT-TO (SUBJAREA, "NEUR")]," reproduced on August 1, 2024, the trends of retractions in medical and health sciences over time, including the countries involved and the funding status, were visualized (Figures 1 and 2).

**Figure 3:**
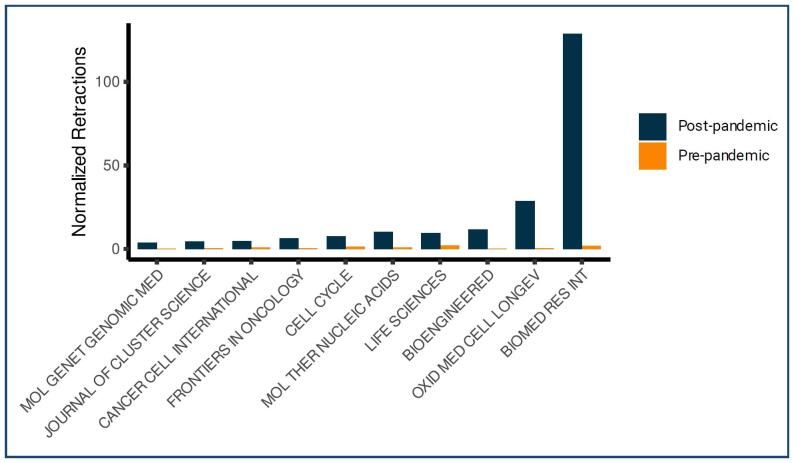
Top 10 journals with significantly increased normalized retraction counts in the post-pandemic period compared to the pre-pandemic period. Normalized retractions are expressed as the number of retractions per year to account for differences in period duration.

The short window for learning in pandemics and the urgent quest for knowledge transformed the COVID-19 theme into not only a highly relevant and necessary field of study but also a coveted topic within the research community. Consequently, numerous ethical committees, grant providers, and journal editors expedited the publication process to ensure rapid dissemination of findings^1^. Living systematic reviews were even updated on a weekly basis^2^. This acceleration came with an inherent need to "balance scientific rigor against speed." Nonetheless, this rapid pace of publication raised valid concerns about the scientific quality of the resulting "*avalanche*." An example is illustrated in Figure 3, which demonstrates a notable increase in retractions in certain journals during the post-pandemic phase compared to the pre-pandemic period.

In a mixed-methods analysis, Zdravkovic et al.^1^ examined 285 PubMed-searched publications from March 12 to April 12, 2020. These publications were divided into COVID-19 (n=155)- and non-COVID-19 (n=130)-associated topics, sourced from the top three scientific journals based on their impact factor (IF)—namely, the New England Journal of Medicine, the Journal of the American Medical Association (JAMA), and the Lancet. The authors assessed the quality of these publications using the "Standard Quality Assessment Criteria for Evaluating Primary Research Papers from a Variety of Fields" (QUALSYST) tool, which assigns a maximum score of 28. The mean total scores for COVID-19 and non-COVID-19 groups were 12.6 (95%CI 10.1–15.1) and 23.7 (95%CI 22.9–24.6), respectively, with a statistically significant difference between the groups (p<0.001; Welch's t-test; Hedges’ g=3.37 and 2.02, respectively). In the same study, it was also observed that the majority of COVID-19 publications fell into the lowest tiers of the evidence pyramid^1^. Specifically, these included cross-sectional studies, opinion papers, case reports, and animal or in-vitro research. Notably, opinion papers and case reports constituted the highest proportion, a trend also highlighted by other authors, concomitant with a substantial number of authors per paper^3^. This is a significant illustration of the value of scientometrics and meta-research in assessing scientific rigor and relevance in biomedical research and scientific publications.

In this context, the "avalanche" has generated doubts about how to assess the relevance and quality of medical research. In recent years, this evaluation has undergone both "formal" and "informal" scrutiny. According to Khokhlov^4^, the former relies on traditional bibliometric indicators such as the IF and the Hirsch index (h-index) of authors and journals. Meanwhile, the latter considers intrinsic quality, studying methodological rigor independent of the traditional indicators. However, there is a growing recognition that relying solely on traditional metrics may not capture the true essence of research quality. Initiatives like the San Francisco Declaration on Research Assessment (DORA) and the Leiden Manifesto for Research Metrics^5^ advocate for a more "informal" approach to assessing research quality. This shift is particularly relevant today, as high IF journals may not always guarantee research excellence. Some authors have raised concerns about the considerable volume of papers being published in journals within short timeframes, prompting questions about the adequacy of peer-review processes^6^.

To ensure scientific rigor in an era of massive low-quality research, the novel field of meta-research involves conducting research on research itself, with the aim of accelerating scientific progress^6,7^. Evidence-based research (EBR) has become a prominent meta-research approach, aiming to generate high-quality and relevant results by systematically and transparently leveraging existing research to inform new studies. EBR serves as a valuable tool to mitigate various pitfalls associated with low-quality research, including non-generalizable results, trivial hypotheses, significant redundancy with existing knowledge, findings that are challenging to disseminate, and interventions that are rarely feasible in practical settings^7^.

In this modern era of science, the approaches of bibliometrics (a branch of scientometrics) and meta-research (the study of research itself) deserve serious consideration. These approaches are valuable not only for assessing research quality but also for informing the production of new research. By embracing these methodologies, the well-known consequences of low-quality research, such as resource misallocation, could be avoided. Such misallocations could disproportionately affect low-income countries of the Global South, where funding and research capacity are limited, ultimately impacting patient health.
